# Atypical Gingivitis: A Rare Entity

**DOI:** 10.7759/cureus.90791

**Published:** 2025-08-23

**Authors:** Ilakiya Mathi, Satarupa Suklabaidya, Kennedy Babu, Soorya K.V., Lakshminarayanan S

**Affiliations:** 1 Periodontology, Mahatma Gandhi Postgraduate Institute of Dental Sciences, Puducherry, IND

**Keywords:** allergens, atypical gingivitis, enlargement, gingiva, gingivectomy

## Abstract

An inflammatory response of the gingiva to particular allergens or an enigmatic cause is known as plasma cell gingivitis (PCG). This atypical form of gingivitis, which involves enlargement of the gingiva in varied degrees, is a sporadic, benign phenomenon.

A 29-year-old female patient presented with painful, bleeding, swollen gums in her upper and lower front teeth region for the past one and a half years. There was no history of parafunctional habits such as mouth breathing, and no history of changing dentifrice recently. Her personal, family, and medical histories did not play a role. On examination, there was enlarged and inflamed gingiva, and a normal complete blood count report. The case was diagnosed as allergic PCG after appropriate investigations and consideration of differential diagnosis. Histopathologic analysis supported the clinical diagnosis. Gingival recontouring was surgically done. To maintain dental hygiene and prevent recurrence, the patient was advised to avoid specific foods. Following a proper histological and clinical diagnosis, a comprehensive treatment plan should be developed.

## Introduction

The human body is full of mysteries, often puzzling clinicians and creating diagnostic and therapeutic challenges. One such condition is plasma cell gingivitis (PCG). It is a non-dental plaque-induced gingival disease characterized by hypersensitivity reactions. This rare condition clinically presents as erythematous, edematous gingiva with loss of stippling and easy bleeding, often extending to the mucogingival junction [1].

Various synonyms used in the literature include atypical gingivitis, idiopathic gingivostomatitis, plasma cell gingivostomatitis, allergic gingivostomatitis, and plasmacytosis of the gingiva [2]. PCG is usually the result of a hypersensitivity reaction to an unknown allergen or antigen, such as flavoring agents like cinnamaldehyde and peppermint oil, found in toothpastes or mouthwashes, artificial flavoring agents in chewing gum, and other food allergens, including spices like chili, pepper, and cardamom [3].

Histologically, the antigen-antibody reaction in PCG is characterized by a marked infiltration of plasma cells within the connective tissue. This report presents a unique case of PCG with no initially identifiable cause. Routine investigations were unremarkable; however, a comprehensive skin prick test involving 120 food allergens revealed sensitivities to certain uncommon food substances. This highlights the importance of thorough allergen evaluation in the diagnosis and management of atypical gingival lesions.

Accurate diagnosis requires a combination of clinical, histopathological, and hematological assessments to distinguish PCG from other clinically similar conditions, including leukemia, atrophic lichen planus, discoid lupus erythematosus, cicatricial pemphigoid, and HIV infection [4].

## Case presentation

A female patient, aged 29 years, had reported to the Department of Periodontics and Implantology at Mahatma Gandhi Postgraduate Institute of Dental Sciences, Pondicherry University, with a complaint of swollen gums and bleeding in the upper and lower front tooth region for the past one and a half years, which was slowly increasing in size over the years and occasionally with burning sensation while taking spicy foods. She reported to the local dentist one and a half years ago, but her problem was not addressed properly. Since then, she had visited various dentists, but the swelling did not resolve with any treatment. Her family history was insignificant. There was no history of known allergies, recent changes in toothpaste or mouthwash, or any parafunctional habits such as mouth breathing.

Personal and medical history

The patient was married and had a four-year-old child. At the time of presentation, she was neither pregnant nor lactating. Additionally, she was not using any hormonal therapies. The patient reported regular menstrual cycles. Her general medical history was non-significant.

Examination

**Figure 1:**
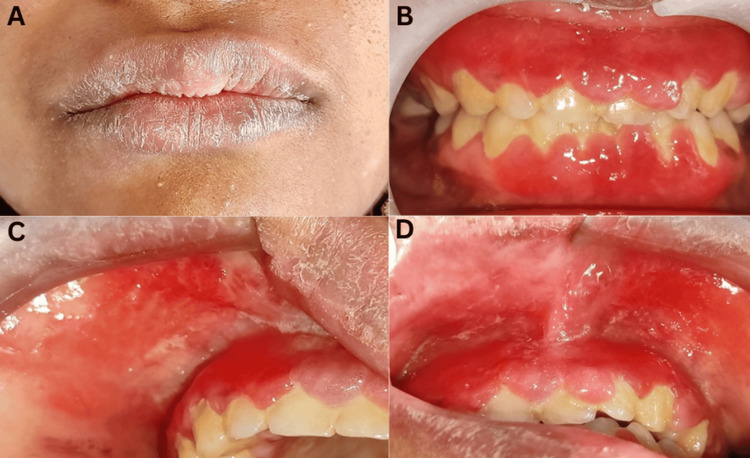
Preoperative images. (A) Swollen and dry lips. (B) Enlarged and erythematous gingiva. (C and D) Erythematous area involving the labial mucosa and vestibule.

Clinically, the gingiva appeared fiery red, enlarged, and erythematous, involving the marginal, papillary, and attached gingiva covering the cervical 1/3rd of maxillary and mandibular anterior from teeth 13 to 23 and 33 to 43, and also extending to the labial and buccal mucosa corresponding to the tooth involved. There was a loss of normal gingival scalloping, with the gingival margins appearing blunt and rolled out. The gingiva was fibroedematous in consistency and bled upon provocation. The condition was also associated with swollen lips. The patient had minimal plaque and calculus with the presence of pseudo pockets in the maxillary and mandibular anterior regions (Figure 1).

Provisional diagnosis

Based on the history obtained from the clinical findings, we provisionally diagnosed it as atypical gingivitis. There were no allergens identified in the past as the cause.

Investigations

There was no evidence of periapical diseases or alveolar bone loss on intraoral periapical radiographs of the upper and lower anterior teeth. Routine blood work was done to assess the overall state of the patient. The results were normal. Excisional biopsy was planned for histopathologic investigation.

Treatment plan

Oral Hygiene Instruction

**Figure 2:**
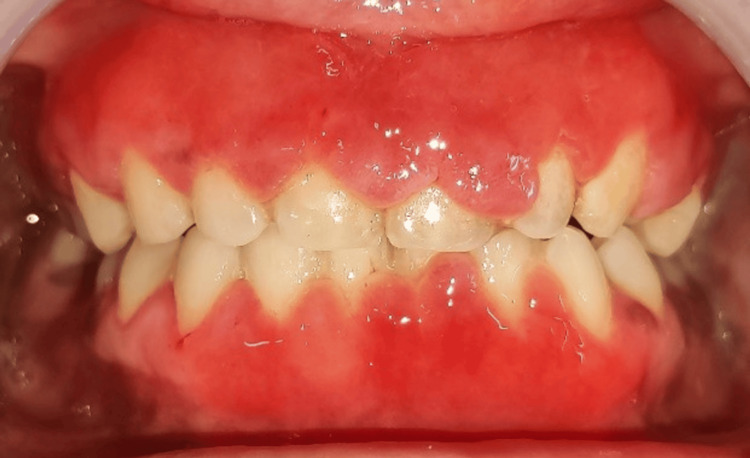
One week after scaling.

The patient was advised to discontinue all forms of toothpaste and mouthwashes and to use only a toothbrush and warm saline gargle twice daily for one week. Scaling was done to remove food deposits (Figure 2).

Surgical Procedure

**Figure 3:**
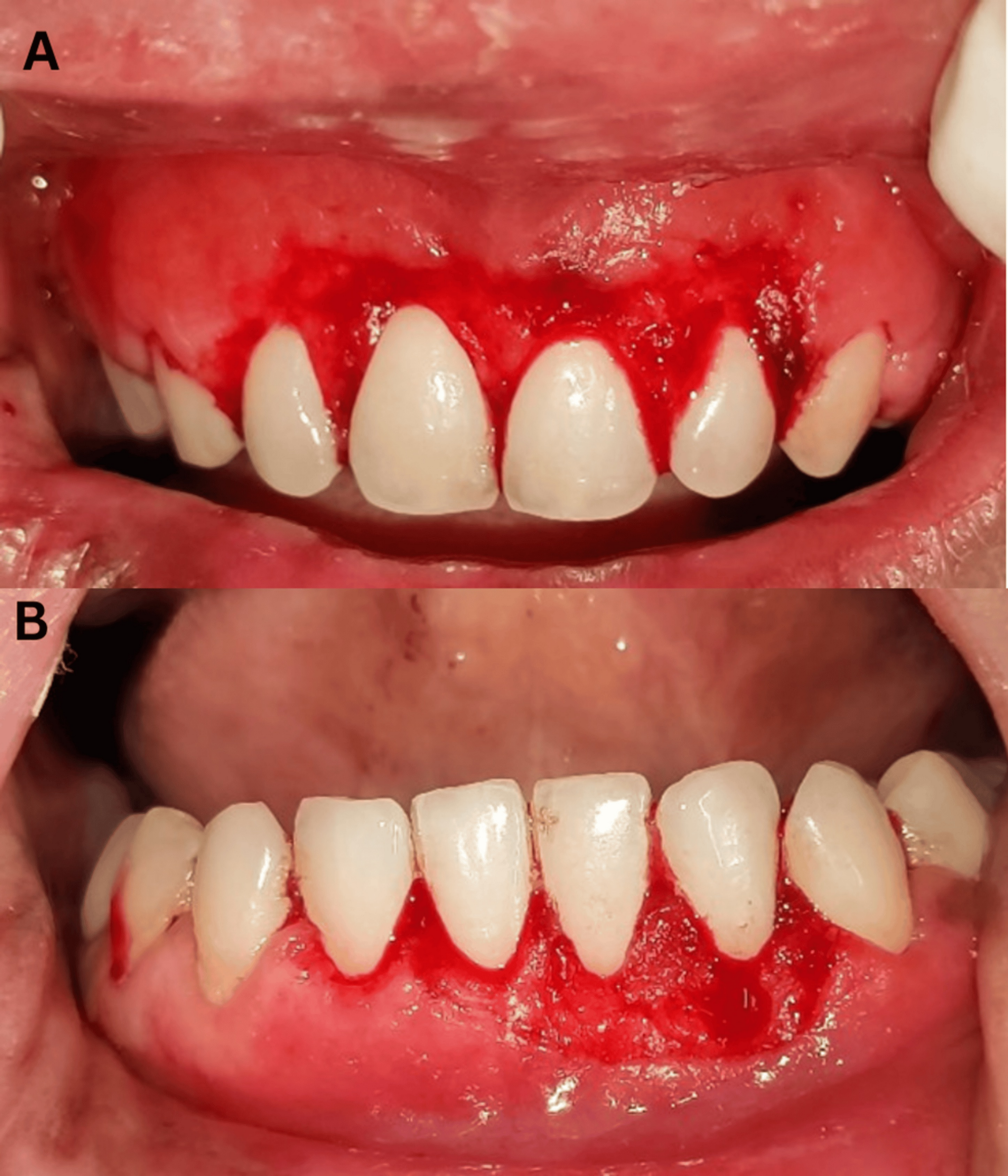
Gingivectomy was done in the maxillary and mandibular anterior regions with a scalpel.

**Figure 4:**
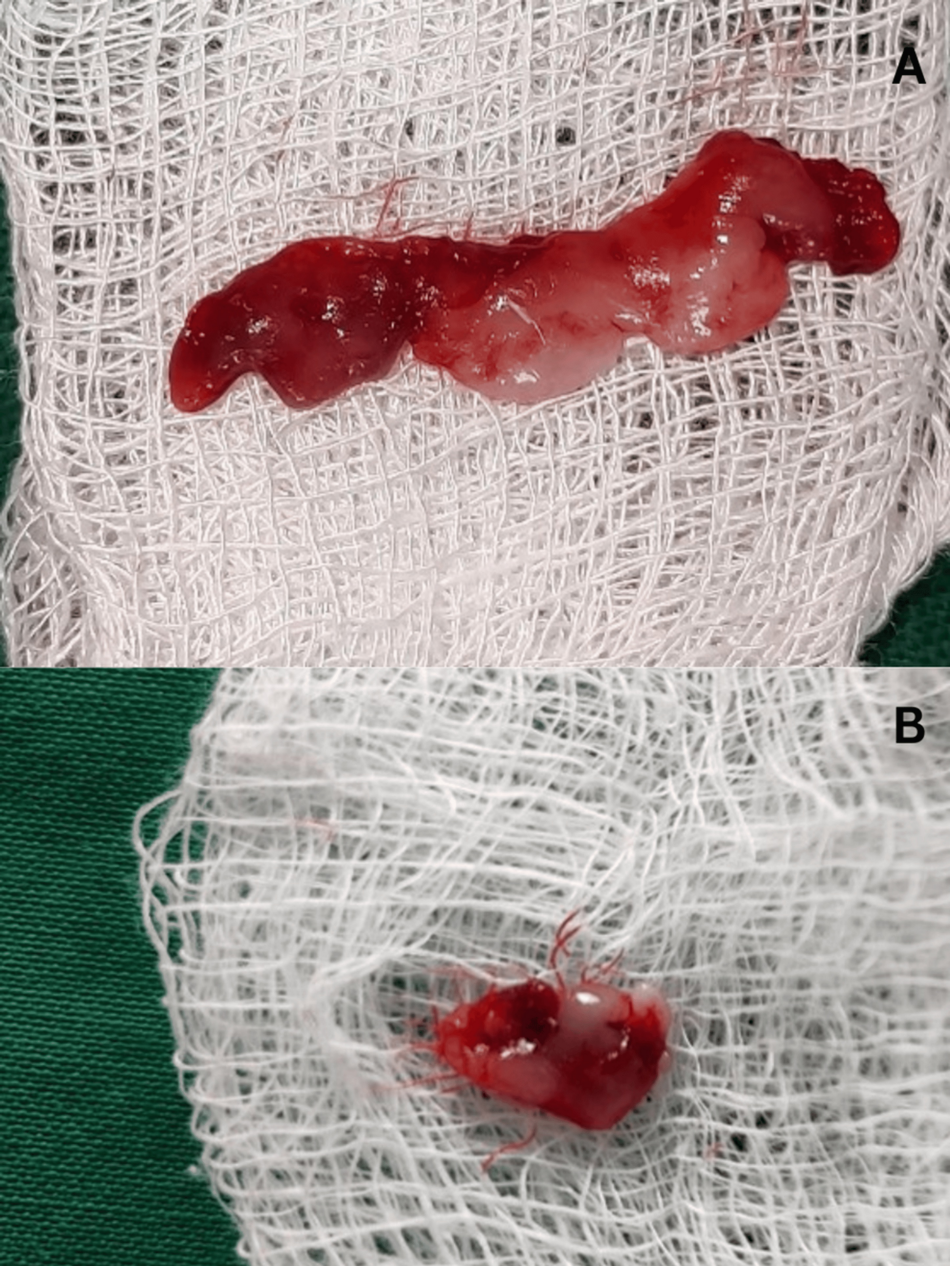
Excised tissue.

Following the attainment of full anesthesia, the alveolar bone level and contour were evaluated using trans-gingival probing. The incision outline was then defined by making marks on the tissue in accordance with the intended clinical crown length. An external bevel incision was created from the markings on the tooth using a No. #15 surgical blade. Crevicular and interdental incisions were made, and a curette was used to remove the tissue. Thus, a gingivectomy was done in the maxillary and mandibular anterior regions with a scalpel (Figure 3). The excised tissues were given for biopsy (Figure 4).

There was no need for osseous re-contouring. Periodontal dressing was applied over the surgical site after hemostasis was achieved. Subsequently, the removed tissue was sent for pathological examination.

Allergens

The patient was tested for nearly 120 food allergens by skin prick test, and found that she was allergic to egg, green tea, tamarind, beans, and brinjal. So she was advised to discontinue these foods.

Histopathologic reports

**Figure 5:**
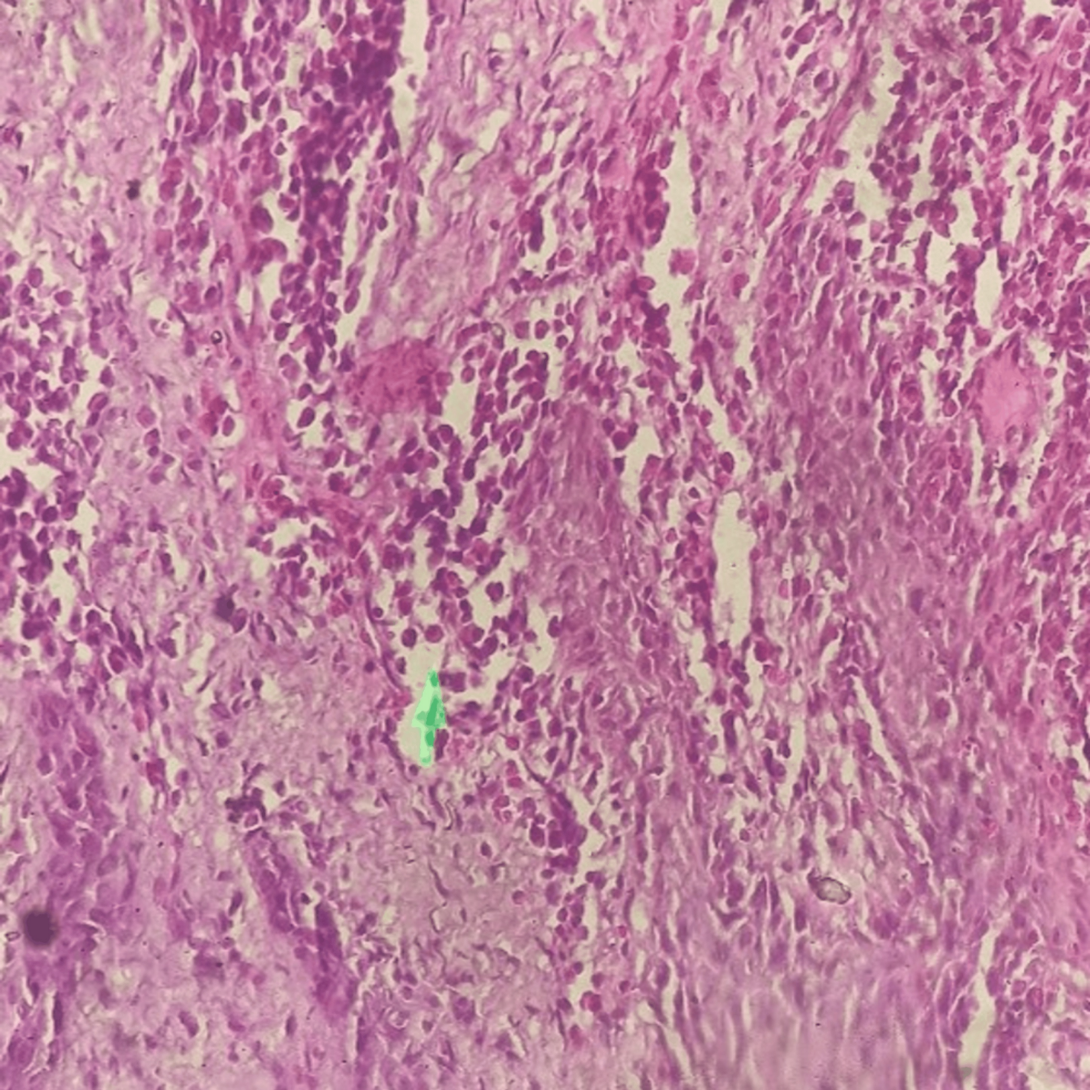
Magnification at 40x showed numerous plasma cells, which exhibited abundant eosinophilic cytoplasm with eccentrically placed nucleus stained using hematoxylin and eosin stain.

**Figure 6:**
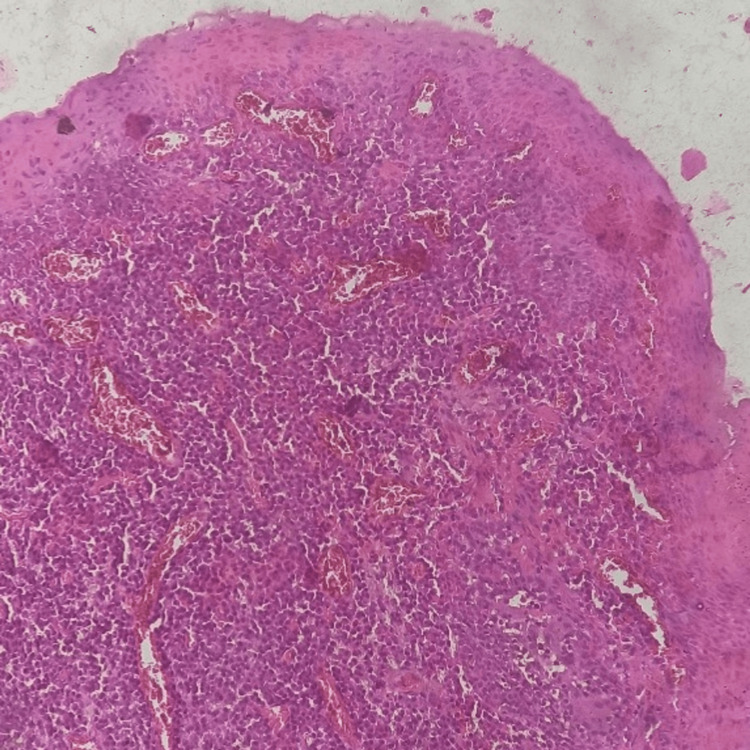
Magnification at 10x showed more of the inflammatory infiltrate, which was stained using hematoxylin and eosin stain.

Histopathological analysis revealed that the stratified squamous epithelium was hyperplastic, acanthotic, and the subepithelial area exhibited a high concentration of persistent inflammatory cells, mainly plasma cells (Figure 5). There was a high density of lymphoplasmacytic cell infiltration in the stroma (Figure 6).

The diagnosis made by histopathologists was gingivitis with a high plasma cell count. The histopathologic findings supported the clinical diagnosis of allergic gingivitis, also known as atypical gingivitis, due to the high concentration of plasma cells in the samples.

Maintenance

**Figure 7:**
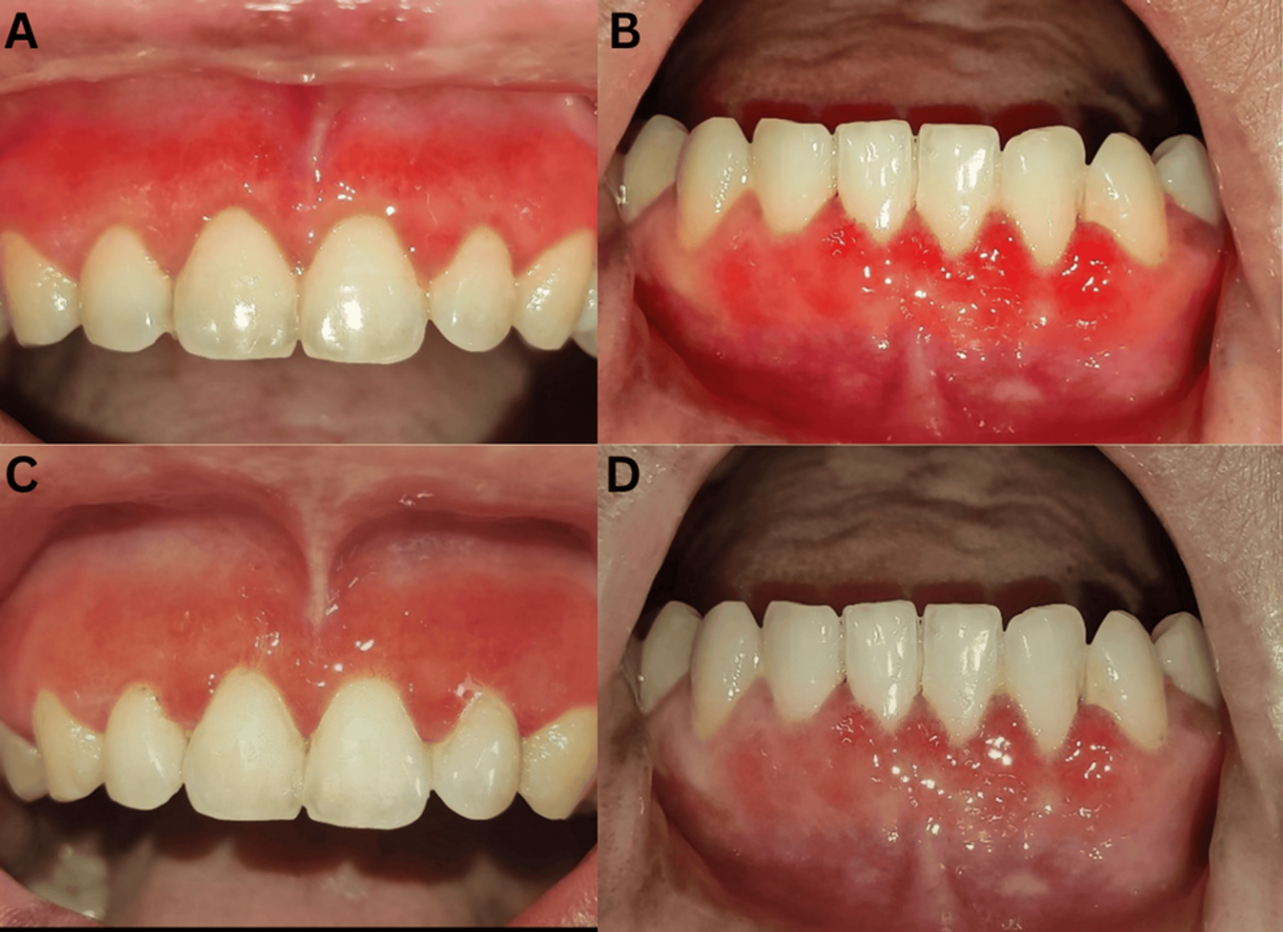
Postoperative images. (A and B) Healing after one month. (C and D) Healing after six months.

The patient was instructed to maintain her oral hygiene exclusively with a toothbrush and toothpaste, and instructed to avoid allergic food substances (beans, tamarind, egg, green tea, and brinjal). The patient was recalled for regular maintenance. Reduction of inflammation was noticed, and the patient was asymptomatic after one and six months postoperatively (Figure 7).

## Discussion

PCG was initially described in 1968 in the United States and has a high prevalence in young females [5]. The benign condition that affects the glans penis was initially reported by Zoon in 1952 under the title "plasma-cell infiltrate" [6]. Additionally, reports of these conditions on the tongue, lips, nasal aperture, epiglottis, conjunctiva, larynx, and vulva have been made [6]. In 1971, Kerr and his colleagues described a case of gingival plasmacytosis and found that chewing gum was the main cause of the allergic reaction [7].

Based on etiology, PCG is categorized into three types: allergic, neoplastic, and of unknown origin [6,8]. The case depicted here is caused by an allergic food substance. This phenomenon is believed to be a disease caused by B cells, to which T cells provide support. PCG is thought to be an infrequent disorder of the gingiva that manifests as a hypersensitivity reaction with abundant plasma cells in the epithelial connective tissue. Nonetheless, the immunologic response to a particular allergenic antigen has been postulated as a potential cause. There is evidence in the literature that chewing khat, chewing gum, mint in toothpaste, cinnamon aldehyde, hot spices, and some ingredients in herbal toothpastes are allergens [6,9]. However, in our case, the identified allergens were different, as we observed food-related triggers such as tamarind, green tea, brinjal, egg, and beans, which are not commonly reported in association with this disease.

Clinically, PCG manifests as a widespread, erythematous, and edematous gingival lesion with a discrete demarcation at the mucogingival junction. It commonly bleeds with touch or slight trauma [8]. Whitened areas, epithelial erosions, and sloughing have been observed [8,10]. In the present case, the gingiva appeared fiery red and enlarged, involving the marginal and attached gingiva, and extending beyond the mucogingival junction to the vestibule, labial, and buccal mucosa. The tissues bled readily on probing despite the minimal presence of plaque. The results of laboratory tests are often within normal bounds, albeit occasionally an increased erythrocyte sedimentation rate is observed. Patch tests to determine an allergy might be useful. Furthermore, a biopsy would be very beneficial in separating various gingival disorders [8]. In the present case, no abnormality was detected in the complete blood picture. Hence, a skin prick test for 120 food allergens was done, and the allergic food substances were constrained to consume. Janam et al. described a case of cheilitis and PCG related to an unidentified allergen, and they used corticosteroids as part of their treatment plan. Nevertheless, the condition did not respond to systemic or topical corticosteroids; thus, surgery was required to treat it [11]. In this case, given the chronic nature of the condition and absence of systemic involvement, a non-pharmacological approach was preferred. Moreover, corticosteroids offer only temporary symptomatic relief and may not prevent recurrence if the underlying allergen is not identified and eliminated. Considering the patient’s favorable response to allergen elimination and oral hygiene improvement, surgical management was sufficient for this patient. Pharmacological immunosuppression was deemed unnecessary. A case study by Joshi et al. described PCG as an allergic reaction to a herbal toothpaste. Here, gingivectomy was used to successfully treat the condition, and even after a year of follow-up, there was no recurrence [12].

## Conclusions

PCG is an interesting condition that is probably allergic in origin. Even if it is totally innocuous, the clinical presentation and location could mask far more harmful illnesses. As a result, every lesion needs to be treated carefully. Excluding all known possible allergens should usually be the first step in first-line therapy, as it may lead to benefits. Improvements in oral hygiene combined with expert periodontal care also typically lead to a decrease in marginal gingivitis. When appropriate, as in this case, excision biopsy of the lesion followed by histological analysis may be the most advantageous course of action in terms of both diagnosis and treatment.
